# The Prevention of Orphanhood

**Published:** 1890-06-14

**Authors:** 


### The Hospital

A Weekly Journal of

Science, Medicine, Nursing, and Philanthropy.

*For Hospitals, Asylums, Medical Practitioners, Students, Nurses, Families, Parishes, Congregations, and the Charitable Public.*

Vol. VIII.—No. 194.] [June 14, 1890.

### THE PREVENTION OF ORPHANHOOD.

The new President of the Royal College of Surgeons, Mr. Jonathan Hutchinson, is a man of original mind, a fact which cannot always be predicated of the presidents of learned societies. He has done what, we venture to say, very few doctors or patients have done: he has asked himself what is the meaning of the word "hospital," and whether that name is the very best which might have been given to a medical charity. He thinks it is not. To himself he always calls a hospital "An Institution for the Prevention of Orphanhood." He does not thereby intend to affirm that all the diseases which are treated at hospitals are dangerous to life, or that those that are dangerous are invariably cured. But he says that many parents are treated at hospitals who would certainly die or be totally disabled but for hospital treatment. He has, therefore, framed his new definition of hospitals, in order that the outside public may, in the first place, have "some adequate conception of the value of those institutions"; and, secondly, may place medical charities in the position which they ought really to occupy—"that of the foremost of all forms of beneficence."

Mr. Hutchinson was the orator at the Mansion House meeting held last week to herald the advent of Hospital Sunday. The hospitals never put forward a better advocate: seldom one so good. In reading his address through for comment, we have found so much that is fresh, so much that is robust, so much that is racy, so much that is reasonable, so much that is poetical and soberly eloquent, that we fear to lay the hand of the commentator upon it lest we should spoil its noble simplicity as a plea for medical charity. "In no other sphere," says Mr. Hutchinson, "can so much good be done at so little cost." A statement like this, if true, demands the attention of all people who give money in charity, whether they give much or little. But is it true? Those who know Mr. Hutchinson well, and who at the same time know intimately the working of the medical charities of Great Britain, will at once say that it is. But many people know neither the working of the medical charities nor Mr. Hutchinson, even though he be the newly-elected President of the Royal College of Surgeons of England.

The new President is well worth knowing. It was very characteristic of him, when he stood on the Mansion House platform last week, that he took it for granted that he ought to present his credentials to his audience. He knew he was going to express opinions, and very decided ones: and he took pains, first of all, to explain to his hearers why he had a right to hold those particular opinions. "I have been," said Mr. Hutchinson, "well behind the scenes, and have known much of the working of not a few institutions. I served my apprenticeship to a gentleman who was a foremost advocate for Provident Dispensaries, and was from my boyhood made familiar with the arguments for and against them. I was in office as *locum tenens* house surgeon to a provincial hospital before I had a diploma and for a year afterwards. At the risk of being accused of pluralism, I may say that after that I was six years at work at the City Hospital for Chest Diseases, more than ten surgeon to the Metropolitan Free, twenty-three on the active staff of the London, during a similar period at the Blackfriars Hospital for Skin Diseases, and almost as long at the Moorfields Ophthalmic. I am familiar both with the out-patients' room and the ward, and know something of the relative systems and Institutions where patients are encouraged to pay towards their expenses, and of those where the aid afforded is always absolutely gratuitous. I hope that in what I have said I have not appeared to be personally boastful. Nothing is further from my wish. I mean simply to justify my right to hold opinions."

Now our constant desire is to hold the scales impartially both for the hospitals themselves and for those philanthropic persons who support them. The hospitals have put forward the President of the Royal College of Surgeons of England as a leading witness before the bar of public opinion. The least instructed reader of newspapers understands that the quality of witnesses is to be taken into account as well as their quantity. Mr. Hutchinson's quality, as judged by the credentials just given, must be considered to be of the first order of excellence. His single arm, like that of Samson, is sufficient to put to flight a whole host of irresponsible and partially-informed newspaper critics. We ask our readers to consider soberly and with British good sense the testimony adduced by Mr. Hutchinson on the various points which have in recent years been urged by hostile critics against hospitals and their management.

The idea that medical charities need to be defended is held by the President of the College of Surgeons to be ridiculous. He tells the story of a Scotch Divine, Dr. McKnight, who wrote a book on the "Harmony of the Four Gospels." A caller at the manse found the good doctor abroad on a visit to his publisher's, and was informed by the man-servant that his master had "gane on a varra feckless errant. He's gane to make four men agree wha never cast oot." To make a defence for hospitals is, in Mr. Hutchinson's estimation, as much a work of supererogation as the harmonizing of the four Gospels. On the whole we are inclined to agree with him. That hospitals in their organization and management sometimes exhibit imperfections he freely admits; but he points out that they are managed by human beings, and that human beings have occasionally been known to err in other departments of life. He says, moreover, that in many directions they are capable of improvement; but he adds that every year improvements are being made. He boldly advances this large general statement, that "On the whole no institutions in the world are better managed than the hospitals of our metropolis. They began," he says, "in the impulses of warm benevolence; they have been nurtured by the efforts of sound judgment; and they are now, it may be, receiving their final development at the hands of a judicious criticism."

There are two or three particular points to which critics adverse to present methods of management usually address themselves. "Indiscriminate charity" is one of them. Mr. Hutchinson argues that medical advice ought not to be spoken of as a form of "indiscriminate charity;" because, he says, "it at least requires of those who receive it that they shall be the subjects of disease or accident." "None of us," he contends, "under such conditions feel the unpaid assistance of others to be either onerous or degrading." With regard to the statements that outs patients are hurriedly and carelessly treated a-hospitals, and that many attend who have no right to be there, he thinks the opinions of the critict cancel one another. For if, he says, it be true that patients desert their own medical men because they can get superior treatment at hospitals, it cannot at the same time be true that hospital treatment is too hurried and careless to be efficient. Other questions under discussion are treated in the same calm and judicial fashion. Indeed, for a clear statement of the case of the Hospitals from their own point of view, there is probably nothing to be found any where equal to Mr. Hutchinson's Mansion House address.

It is impossible in a brief leading article to do full justice to this carefully-reasoned argument and appeal. Still less does space permit us to enter upon questions on which we differ from the judgment expressed. We prefer, indeed, to forego adverse criticism altogether, and to press upon our readers what Mr. Hutchinson so ably urged upon his hearers at the Mansion House. He asked for the intelligent sympathy of his audience. "What is necessary," he said, "is to increase our experience. Let anyone who is conscious of lack of sympathy with the afflicted go for a week to his usual avocations with a black patch covering one eye; let him wear for one day a wooden leg, a truss, or a spinal apparatus; let him choose some leisure day in the country in bright spring, and resolutely for twenty-four hours keep a bandage firmly placed over both eyes. His organization is, I fear, well nigh hopeless if he does not feel inclined next morning to send a liberal donation to some hospital. Yet let me remind him that what he has realized has been only the inconvenience of a temporary loss of sight, and that he has tasted not at all of the horror of the thought, 'For me there is only the blackness of darkness to the end of life.'" Hospitals will do well to publish Mr. Hutchinson's noble address, and not only to send it to every member of the Lords' Committee on Hospitals, but also to every subscriber, and to every man of means and position throughout the country. A worthier vindication of our voluntary medical charities has never been penned. We have therefore arranged, with Mr. Hutchinson's sanction, to publish it separately for general distribution in a cheap form.

